# Endophytic bacteria of *Fagonia indica* Burm. f revealed to harbour rich secondary antibacterial metabolites

**DOI:** 10.1371/journal.pone.0277825

**Published:** 2022-12-15

**Authors:** Lubna Rahman, Asma Mukhtar, Sajjad Ahmad, Lutfur Rahman, Muhammad Ali, Muhammad Saeed, Zabta Khan Shinwari

**Affiliations:** 1 Department of Biotechnology, Quaid-i-Azam University, Islamabad, Pakistan; 2 Department of Chemistry and Chemical Engineering, SBA, School of Science and Engineering LUMS, Lahore, Pakistan; 3 Department of Health and Biological Sciences, Abasyn University, Peshawar, Pakistan; 4 National Institute for Biotechnology and Genetic Engineering (NIBGE), Constituent College Pakistan Institute of Engineering and Applied Sciences (PIEAS), Islamabad, Pakistan; 5 Department of Plant Sciences, Quaid-i-Azam University, Islamabad, Pakistan; Tocklai Tea Research Institute, INDIA

## Abstract

Endophytic bacteria are the source of novel bioactive compounds, used as therapeutic agent. Molecular docking is a computational technique use frequently, to find novel drugs targets and drugs-receptors interactions. The current study was designed to isolate and identify endophytic bacteria for the extraction of bioactive compounds. Further, to characterized extracts and to explore compounds interactions with bacterial cell wall and outer membrane synthesizing proteins. Endophytes were identified using 16s rRNA amplification technique. For bioactive compounds, solvent extraction method was followed and characterized further through GC-MS analysis. To find targets and drugs-receptors interactions, molecular docking studies and biological assays were performed. The isolated endophytes belong to five different genera namely *Enterobacter*, *Bacillus*, *Erwinia*, *Stenotrophomonas* and *Pantoea*. In case of antibacterial assay *Stenotrophomonas maltophilia* extract showed significant inhibitory zones (15.11±0.11mm and 11.3±0.16) against *Staphylococcus caseolyticus* and *Acinetobacter baumanni*, with MIC 33.3 and 50μg/mL respectively. Among the characterized fifty compounds, from endophytic bacteria “antibacterial compound” N-(5-benzyl-10b-hydroxy-2-methyl-3,6-dioxooctahydro-8H-oxazolo[3,2-α] pyrrolo[2,1c] pyrazin-2-yl)-7-methyl2,3,3a,3a^1^,6,6a,7,8,9,10,10a,10b-dodecahydro-1H-4λ^2^-indolo[4,3-*f*g]quinoline-9-carboxamide of bacteria *Stenotrophomonas maltophilia* were an excellent binder with MurF ligase active site, with binding energy of -10.2 kcal/mol. Extracts of endophytic bacteria composed of various pharmacologically active ingredients such as antibacterial compounds. Molecular docking studies provide important information regarding drug-receptor interaction, thus can be used in novel drug discovery.

## Background

Biologically active compounds obtained from natural resources have significant role in the field of biomedicines and disease treatment. Natural resources include plants, animals, and their associated microbes (Bacteria, Fungi and actinomycetes) as well as those microorganisms living in different environments such as algae, corals and sponges [1]. These biomolecules are considered more effective medicines, as compared to synthetic and semisynthetic compounds due to their biocompatible nature, relative abundance, and negligible side effects [2].

Secondary metabolites are broadly classified into three main groups; terpenes (carotenoids, cardiac glycosides, plant volatiles and sterols), phenolics (flavonoids, lignans, lignin, tannins coumarins, stilbenes and phenolic acids) also include alkaloids and glucosinolates (nitrogen containing compounds). It has been reported that the synthesis of medicinally important secondary metabolites (SM) occurs mostly in the early hours of the stationary phase of microbial growth, thus their real time monitoring needs to be optimized. Investigation of endophytic based SMs can provide a new platform for the manufacturing of potent natural compounds against various diseases [3, 4]. For a wide range of biological activities endophytic metabolites have been evaluated such as neuroprotective, antimicrobial, cytotoxicity, immunosuppressive, anti-parasitic, enzyme inhibitive and insulin-mimetic [5]. A single strain of endophyte can synthesize a large number and different classes of bioactive secondary metabolites. In this regard, their identification for the sake of purification and further biomedical applications is very much important. Up to date, numerous traditional procedures have been employed for the separation and identification of SMs each analytical technique depending on the nature (volatile, nonvolatile) and class of compounds as well as on the organism. During the extraction process of secondary metabolites, selection of solvent (i.e., based on polarity) is also an important parameter. Moreover, to facilitate SMs extraction, sonication, microwave treatment, shaking and temperature also have significant role. For the characterization and quantification of such targeted or novel SMs, Gas Chromatography-Mass Spectrometry (GC-MS) is considered as one of the modern and reproducible analytical methods. The basic principle of the GC/MS technique is to separate individual components of crude extract, consisting of many compounds with high sensitivity, specificity and selectivity. Further a minute level contaminant detection, classified and structural characterization are a few useful applications of GC/MS analysis [6].

For novel drug discovery from natural resources, characterization plays a key role. Similarly, there is the need to develop and use special techniques and strategies, to study the interactions between the ligand (i.e., compounds) and targeted entities in the biological systems. The integration of computational and experimental approaches can provide a baseline in the field of drug discovery. Thus, molecular docking is a useful technique to find ligand-target integrations prior to experimental conduction to save resources. The molecular docking was first time developed in 1980. Where Kuntz et al. (1982) introduced an algorithm designed to fix macromolecular receptors into their small molecules [7, 8]. Molecular docking is a powerful approach in structure-based drug designing. The main objective of this technique is to identify new active compounds for a particular targeted protein. It works on different approaches such as monte carlo, matching approach, ligand fit approach, point complimentarily approach, fragment based, distance geometry based, and inverse docking etc. The molecular docking technique can be used in drug molecule (hit) identification, lead optimization and remediation. Molecular docking is an extensively used strategy due to its simplicity, low-cost and advanced predictability. The molecular docking mechanism is two steps process: prediction of ligand conformation with respect to receptor and estimation of binding affinity (usually in kcal/mol [9].

The current study aims to characterize secondary metabolites, extracted from endophytic bacteria, isolated from *F*. *indica*, using GC-MS analysis. Moreover, the antimicrobial activity of the extracts was investigated. To further affirm and support the experimental findings, computational docking and simulation were performed. Such computational and experimental assays performed may aid in unveiling the real affinity of the compounds for several antibacterial targets. It may also guide and convince researchers to perform further structure optimization of the high-affinity binders. The overall study showed that isolated endophytic bacteria are a dependable source of bioactive compounds having the significant potentials of antibacterial efficacy. Molecular docking result revealed that the compounds show affinity and holds a promising lead target toward the broad-spectrum receptor proteins involved in the biosynthesis of pathogenic bacterial cells outer membrane.

## Material and methods

### Endophytic bacteria isolation, molecular identification and secondary metabolites extraction

Endophytic bacteria were isolated from *Fagonia indica* Burm.f following previously reported protocol [10]. Briefly, the plants were collected and cut into small pieces (0.5-1cm) in length. It was then surface sterilized with 70% ethanol and commercial bleach (Clorox). To remove all the traces of Clorox, the obtained plant pieces were washed with autoclaved distilled water. The washed pieces were blotted on the sterilized blotting paper to remove water. Finally, the pieces were placed on Tryptic Soy Agar (TSA) media plates for bacterial growth. The plates were incubated at 30°C for 24 h to obtain different colonies of bacteria. The bacteria were streaked several times to obtain pure bacterial colonies. Afterward pure culture of single bacterial colonies was isolated and processed further for identification through 16s rRNA gene amplification with 27F and 1492R universal bacterial primers through colony PCR. PCR products were purified with LinkTM Quick PCR purification Kit from Invitrogen and Sanger sequencing was done commercially from Macrogen (South Korea). Sequences were submitted after blasting in EZ-taxon (http://www.ezbiocloud.net/eztaxon) and NCBI (www.ncbi.nlm.nih.gov/BLAST) databases. The nucleotide sequences were deposited in GenBank for the isolation of accession numbers. Furthermore, the phylogenetic tree was constructed using isolates sequences and reference sequences from the NCBI database and EZ-taxon. The analysis was conducted with Molecular Evolutionary Genetics Analysis software 7.0 (MEGA 7.0) and BioEdit (version 5.0.6) using neighbor-joining method (Bootstrap analysis with 500 replicates).

### Secondary metabolites isolation

For the extraction of secondary metabolites protocol previously, described by [10] was used with some modification. Tryptic soy broth was prepared in flask, autoclaved, and used for the endophytic bacteria inoculation. After inoculations flasks were incubated in shaking incubator at 30°C for 48 hrs at 120 rpm. After incubation the bacterial inoculation was transferred to 50mL falcon tubes and followed by centrifugation for 10 mins at 10,000 rpm. Supernatant was discarded and the pellet was processed further for the isolation of secondary metabolites. Pellet was dissolved in organic solvent (methanol) and incubated for 2 to 3 hrs. after incubation to rapture the bacterial cell sonication was performed 3 to 4 times for 30 mins after every 5 mins. After completion of the sonication the tubes were again centrifuged at 10,000 rpm for 10 mins. The organic solvent was collected, and the pellet was discarded the process was repeated two time. Organic solvent (Methanol) was evaporated under reduced pressure on BUCHI Rotavapor at temperature <50◦C and the remaining extract of bacterial secondary metabolite was dissolved in dimethyl sulfoxide (DMSO) for further used.

### Antibacterial assay

Antibacterial activity of endophytic crude extracts was investigated against pathogenic Gram-positive strains *Staphylococcus epidermis* ATCC 35984, *Staphylococcus aureus* ATCC 25923, *Staphylococcus caseolyticus* ATCC 13548, Methicillin-resistance *Staphylococcus aureus* ATCC 33591 and Gram-negative multi drugs resistant strains (MDRs) *Enteriobactor cloaca* ATCC 13047 and *Acinetobacter baumannii* ATCC 17978 through well diffusion method as described previously [11]. For the assay, Tryptone soy agar (TSA) media was prepared and autoclaved at 121°C for 20 mins and poured into sterilized petri plates under antiseptic conditions. Tryptone soy broth (TSB) 10 ml was used for the pathogenic bacterial strains inoculation and was incubated for 24 hrs at 37°C with continuous shaking (120rpm). Suspension of each bacterial strain was prepared, and turbidity was adjusted as compared to 0.5 McFarland solution (1.5×10^8^ CFU/mL) at 600 nm. The bacterial culture was then distributed uniformly on TSA plates using sterile cotton swabs, followed by the formation of 10mm wells at a specific distance from each other. Stock solution of each bacterial extract was prepared in Dimethyl sulfoxide (DMSO), labeled and calculated concentration was added to the respective well (n = 3). DMSO, ampicillin and meropenem (positive control of MDR strains *Enteriobactor cloaca* and *Acinetobacter baumannii*) were used as negative and positive controls respectively. We incubated the plates at 37°C for 24 hrs and zones of inhibition were measured for each sample (n = 3) and active samples were further proceeded for MIC determination [12].

### Quantitative analysis

#### Gas chromatography- mass spectroscopy (GC-MS)

The samples were analyzed for the detection of compounds through Gas Chromatography- Mass Spectroscopy (GC-MS). The compounds were separated by using (GC, Trace-1300, Thermo scientific) and capillary column (TR-35 MS) with (30 m x 0.25 mm I.D. x 0.25 μm) dimensions. For the separation of compounds Helium gas was used. The temperature of the injection port was maintained at 250°C while 1 μL of the sample volume was injected for compound separation. The initial temperature of column was maintained at 50°C for 1 minute, and then programmed at 20°C/min to 300°C for 38 min. The known compounds stored in the GC-MS library and software data system were used for comparison of the results spectrums of separated compounds.

### Statistical analysis

All the experiments were conducted in a triplet manner and to assess the relationship between different parameters Pearson’s correlation coefficient (r) was used. Origin 8.1 was used for all the graphs. While for statistical analysis SPSS 22.0 and Statistic 8.1 was used with Tukey’s HSD for post hoc analysis. The significant mean difference was calculated using one-way ANOVA at P < 0.05 and 0.001.

### Molecular docking

After quantitative analysis of bacterial metabolites, the compounds identified with help of GC-MC library and, Data System (CDS) software were further used for Molecular docking studies to determine the affinity of these compounds against the different broad-spectrum receptor proteins of pathogenic bacteria.

#### Molecular docking for antibacterial affinity

The 2D structure of all compounds was drawn in ChemDraw Ultra 12.0 (ChemOffice package), followed by ensuring proper orientation and correctness of bond order [13]. The molecules were then converted to 3D and subjected to energy minimization through MM2 force field. Broad-spectrum receptor proteins involved in the biosynthesis of the bacterial cell, outer membrane and were chosen for docking studies. These include KdsA, KdsB, KdsC (take part in the synthesis pathway of KDO sugar molecule in gram-negative bacterial lipopolysaccharide), MurC, MurD, MurE, MurF (key enzyme in the biosynthesis of bacterial cell wall of both Gram- negative and positive), D-alanine-D-alanine ligase (take part in bacterial peptidoglycan biosynthesis), RstA and KdpE (bacterial two component system proteins). The crystal structure of the mentioned proteins was retrieved from protein data bank (PDB) and prepared in UCSF Chimera version where any associated ligand molecules, as well as water molecules, were removed. Each protein was also minimized for 1000 rounds (500 steepest descent and 500 conjugate gradient steps) [14]. Both the receptors and the compounds were then used as input for docking studies in AutoDock 4.2. Docking simulation was performed through “AutoDock tools” graphical user interface program where polar hydrogens, solvation parameters and Kollman charges were added to the proteins. The grid was set as such to cover the complete surface of the proteins and allow the ligands to bind to the region where they are showing the maximum affinity. For each compound, 10 conformers were generated using Lamarckian Genetic Algorithm (LGA). The docking predictions were repeated in triplicate and the mean value of the scores was considered as the binding affinity of the compounds. The top complex for each compound based on the docking score was selected and visualized and analysed in UCSF Chimera and Discovery Studio [15].

#### Molecular dynamics simulation

The top complex dynamics were unveiled using simulated annealing with NMR-derived energy restraints (SANDER) module of AMBER18 Parameter files for the protein receptor and the ligand molecule using ff14SB [16] and general amber force field (gaff) [17] respectively. The complex was then solvated in TIP3P water box with 12 Å distance between the complex and water box edge. By adding an appropriate number of counterions, the net charge of the system was neutralized. System minimization was achieved by subjecting the system to 2000 rounds comprising 1000 steps of conjugate gradient and 1000 steps of steepest descent. The non-bounded interactions cutoff was allowed to 8 Å. System heating was done for 100 ps by slowly moving the temperature from 0 K to 100 K keeping the pressure of 1 atm. Following this, the system was equilibrated for 100 ps at a temperature of 300 K. A production run of 100 ns was performed for the system where long-range interactions are treated by Ewald summation. The PTRAJ module [18] was lastly used to statistically analyze the dynamics of the system through several different assays.

#### MMPBSA binding free energies

The intermolecular chemical interactions and the strength of association between the ligand molecular and the receptor MurF protein were determined using an end point MM-PBSA method of AMBER18 [19].

The net binding energy for the system was predicted using Eq (i),

$$\Delta \text{G}=\Delta \text{G}\;\text{complex}–\left(\Delta \text{G}\;\text{receptor}–\Delta \text{G}\;\text{ligand}\right)$$ (i)

ΔG in each equation represents Gibb’s free energy and can be split into,

ΔG=Egasphaseenergy+ΔGsolvationenergy–TSsolute (ii)

Where T stands for temperature and S for entropy contribution to ligand binding.

## Results

### Endophytic bacteria isolation, molecular identification, and phylogenetic analysis

The isolated eight endophytic bacteria from *F*. *indica* labeled as MOSAEL [FIS1(*Fagonia indica* stem)-FIS8] with (5.78 × 101 CFU/mL) were identified through 16S rRNA gene sequence analysis. Selected strains were identified as belonging to different genera namely *Bacillus*, *Enterobacter*, *Pantoea*, *Erwinia* and *Stenotrophomonas*. The sequences were submitted in NCBI and accession number from KT367786 to KT367793 were obtained for the respective strains of bacteria. Neighbor-joining method (Bootstrap analysis with 500 replicates) was used to generate phylogenetic tree through MEGA-X software by using the partial 16S rRNA gene sequences of the putative isolated endophytic bacterial with representative bacterial strains of related taxa from NCBI database (S1 Fig).

### Antibacterial assay

Zones of inhibition against the tested pathogenic bacterial strains were measured and compared with positive controls (Ampicillin) (S2 Fig) Results demonstrated that the endophytic bacterial extracts revealed broad-spectrum antagonistic potential (Table 1). Where *S*. *maltophilia* (FIS2) represents remarkable antibacterial potency toward *S*.*caseolyticus* and *A*.*baumanni* with 15.2±0.11 mm and 11.3±0.16mm zones of inhibitions having 33.3,50μg/ml MIC respectively. *B*. *taquilensis* (FIS3) has been found more active toward the gram-negative strains (*E*. *cloacae* and *A*. *baumanii*) with 10.4±0.26 and 12.6±0.12 mm zone of inhibition at 33.3 and 50μg /mL MIC respectively. Furthermore, *P*. *dispersa* (FIS5) exhibited a minimum Zone of inhibition against *S*. *aureus*, *E*. *cloacae*, *A*. *baumanii*, and *S*. *caseolyticus* having (50 and 100μg/mL MIC) (Table 2).

**Table 1:** Antibacterial activity of bacterial crude extracts of medicinal important plant (*Fagonia indica*) against Gram- positive and Gram- negative human pathogenic strains. Each bacterial extract was analyzed individually in triplicate (n = 1 × 3) where (mean ± SD) are average of each value.

Pathogenic bacteria													
Entophytic bacteria bioactive secondary metabolites		*Staphylococcus epidermis*		*Staphylococcus Aureus*		*Staphylococcus caseolyticus*		*Methicillin-resistance Staphylococcus aureus*		*Enteriobactor cloacae*		*Acinetobacter baumannii*	
S.no	Endophytic bacteria	Zone of inhibition in mm	MIC μg/mL	Zone of inhibition in mm	MIC μg/mL	Zone of inhibition in mm	MIC μg/mL	Zone of inhibition in mm	MIC μg/mL	Zone of inhibition in mm	MIC μg/mL	Zone of inhibition in mm	MIC μg/mL
FIS1	*E*. *hormaechei*	9.7±0.25**	100	10.6±0.40	100	8.5±0.20**	100	10.5±0.20**	200	10.1±0.15	100	8.2±0.25**	100
FIS2	*S*. *maltophilia*	10.3±0.26**	100	10.4±0.26	50	15.2±0.11**	33.3	10.3±0.25**	100	11.4±0.35	33.3	11.3±0.16**	50
FIS3	*B*. *tequilensis*	9.4±0.34**	100	11.2±0.31	100	8.5±0.25**	100	9.2±0.15**	50	10.4±0.26	100	12.6±0.12	33.3
FIS4	*Erwinia sp*.	9.1±0.30**	200	9.1±0.41	200	10.2±0.16**	150	8.4±0.26**	200	6.2±0.26**	100	9.4±0.32**	200
FIS5	*P*. *dispersa*	8.6±0.15**	200	10.1±0.41	50	12.9±0.10**	50	7.2±0.25**	200	10.9±0.15	100	9.4±0.26**	100
FIS6	*P*. *cypripedii*	7.5±0.39**	200	11.2±0.20	100	7.4±0.50**	100	8.8±0.51**	250	9.8±0.50	100	8.2±0.21**	100
FIS7	*E*. *cloacae*	8.5±0.20**	100	10.4±0.26	100	7.4±0.27**	250	6.4±0.35**	200	8.5±0.40*	100	5.5±0.40**	100
FIL 8	*B*.*subtilis*	7.5±0.36**	100	9.3±0.25	200	8.5±0.18**	200	7.6±0.43**	150	8.6±0.25*	100	6.4±0.41**	100
P.C	Ampicillin	20.3±0.26	-	11.4±0.10	-	32.6±0.30	-	27.4±0.25	-		-		
P.C	Meropenem									10.5±0.95		-	-
												13.5±0.80	
N.C	DMSO	-	-	-	-	-	-	-	-	-	-	-	-

Where two star (**) shows highly significant (p-value<0.001), one star (*) shows significant p-value<0.05 and no star shows non-significant values.

**Table 2:** The least significant differences (LSD) to compare means of activated samples with control group.

*Staphylococcus Epidermis*	*Staphylococcus Aureus*	*Staphylococcus Caseolyticus*	*Methicillin-resistance Staphylococcus Aureus*	*Enteriobactor Cloacae*	*Acinetobacter baumannii*
10.43333**	0.80000	24.13333**	16.90000**	0.73333	5.30000**
10.00000**	1.00000	17.40000**	17.10000**	0.10000	3.20000**
10.90000**	0.20000	24.10000**	18.20000**	0.10000	0.90000
11.23333**	1.96667	22.46667**	19.00000**	4.30000**	4.10000**
11.70000**	1.30000	19.70000**	20.20000**	0.40000	4.10000**
12.80000**	0.30000	25.20000**	18.60000**	0.70000	4.96667**
11.80000**	1.00000	25.20000**	21.00000**	2.00000*	8.00000**
12.80000**	1.76667	24.10000**	19.80000**	1.90000*	7.10000**
**F = 24.487**	**F = 0.603**	**F = 124.429**	**F = 102.541**	**F = 5.745**	**F = 27.826**
***p-value* = 0.000**	***p-value* = 0.763**	***p-value* = 0.000**	***p-value* = 0.000**	***p-value* = 0.001**	***p-value* = 0.001**

Where two star (**) shows highly significant (p-value<0.001), one star (*) shows significant p-value<0.05 and no star shows non-significant values.

### Quantitative analysis

#### Gas chromatography -mass spectroscopy (GC-MC)

**Fig 1:**
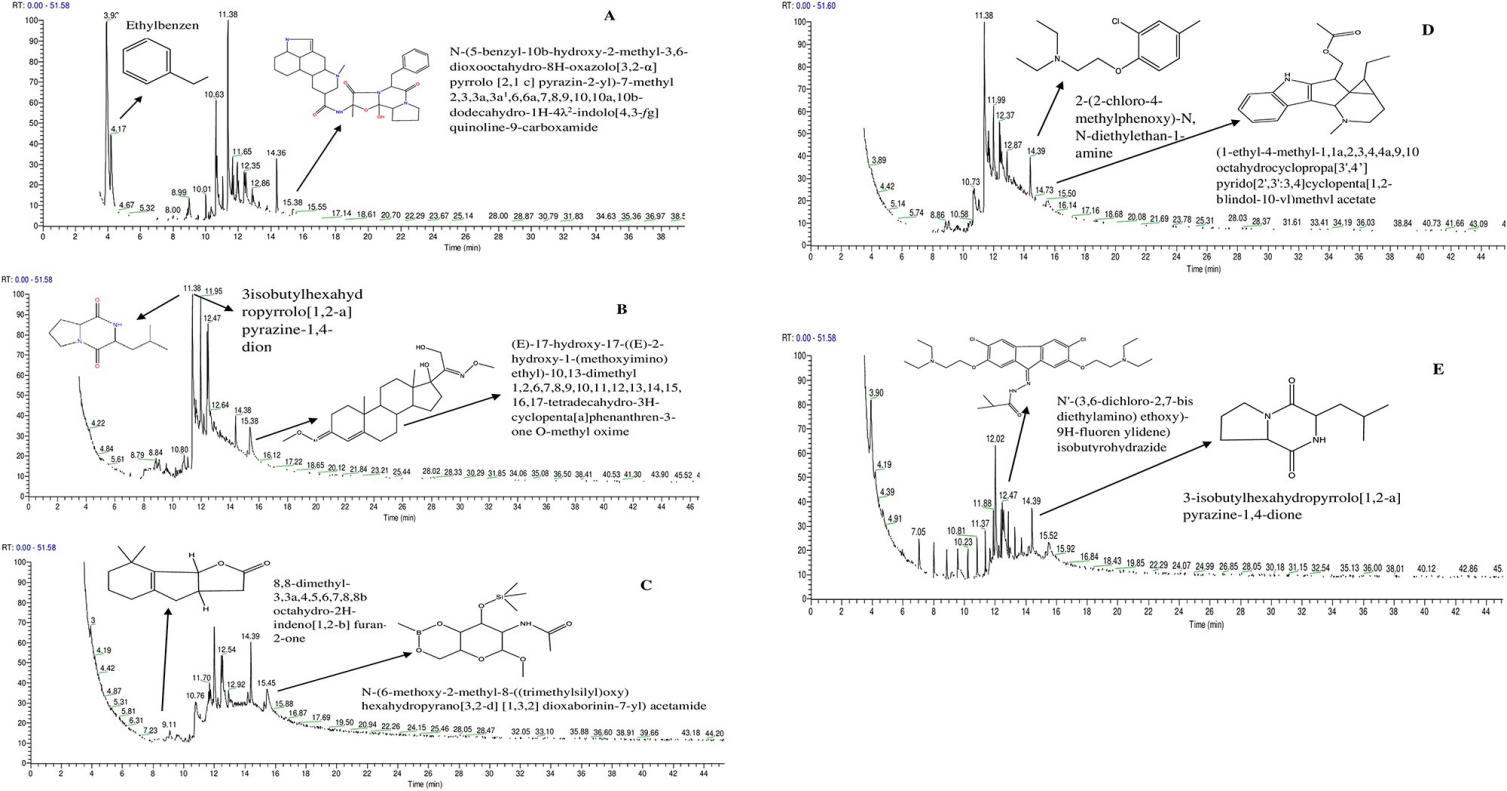
**(A-E)** Total ion chromatogram (TIC) of the methanolic extract of endophytic bacteria of *Fagonia indica*, represents the GC-MS profile of the identified compounds. Where **(A)** represent *S*. *maltophilia*, **(B)** *B*. *tequilensis*, **(C)** *P cypripedii*, **(D)** *E*. *cloacae*, and **(E)** *B*. *subtilis*.

The preliminary quantitative analysis of bacterial metabolites GC-MS leads to the identification of several secondary class compounds. The total ion chromatogram (TIC) of the methanolic extracts, showing the GC-MS profile of the compounds identified is given in (Fig 1).

The spectrum of GC-MS with different retention times revealed the presence of various components. The GC-MS spectrometer analyzes the compounds nature and structure which eluted at different times (S3 Fig). GC-MS analysis determined the presence of different alcoholic, fatty acids, heterocyclic and esters among other compounds when large compounds fragments incorporated into small compounds giving rise to the appearance of peaks at different ratios time. The X calibur software data system and Library systems from NIST (National Institute of Standards and Technology) were used to analyze the obtain mass spectra of separated compounds. The NIST/ EPA/ NIH main library (mainlib) was used to analyze and match the data where 50 different compounds from bacterial secondary metabolites were characterized out of which 6 compounds were identified from *E*. *hormaechei*, 11 from *S*.*maltophilia*, 7 from *B*. *tequilensis*, 4 from *Erwinia spp*, 3 from *P*. *dispersa*, 7 from *P*. *cypripedii*, 8 from *E*. *cloacae* and 4 from *B*. *subtilis*.

The active constituents with their molecular weight (MW), retention time (RT), concentration (peak area %) and Molecular formula (MF) were found in (S1 Table) where Bis (6-methylheptyl) phthalate (45%), (E)-hexadec-4-enal (56%), p-xylene (87.3%), Pentadecanoic acid (44.4%), n-Hexadecanoic acid (36%),2-((2-chlorocyclohexyl)oxy)-N,N-diethylethan-1-amine (62.5%),N’-(3,6-dichloro-2,7-bis(2-(diethylamino)ethoxy)-9H-fluoren-9 ylidene) pivalohydrazide (71.4%), 3a-methyl hexadecahydro cyclopenta[b]fluoren-3-ol(51.3%),N-(6-methoxy-2-methyl-8 ((trimethylsilyl)oxy)hexahydropyrano[3,2d][1,3,2]dioxaborinin-7-yl)acetamide(80%) and 3-isobutyl hexahy dro Pyrrolo [1,2-a]pyrazine-1,4-dione (65.6%) were obtained as major components. It is notable that in metabolites, the major constituents were identified, while the compounds with the highest composition have not been matched in the library which could be novel and need to be analyzed further to elucidate their nature. GC-MS results show that the compounds present in the bacterial metabolites include major constituents like esters, fatty acids, alcohols, aldehydes, terpenes and amide etc. Compared with the antimicrobial results this entire constituent shows promising antimicrobial activity especially antimicrobial lipids (Fatty acids and monoglycerides) as reported previously [20].

#### Molecular docking with antibacterial targets

**Fig 2:**
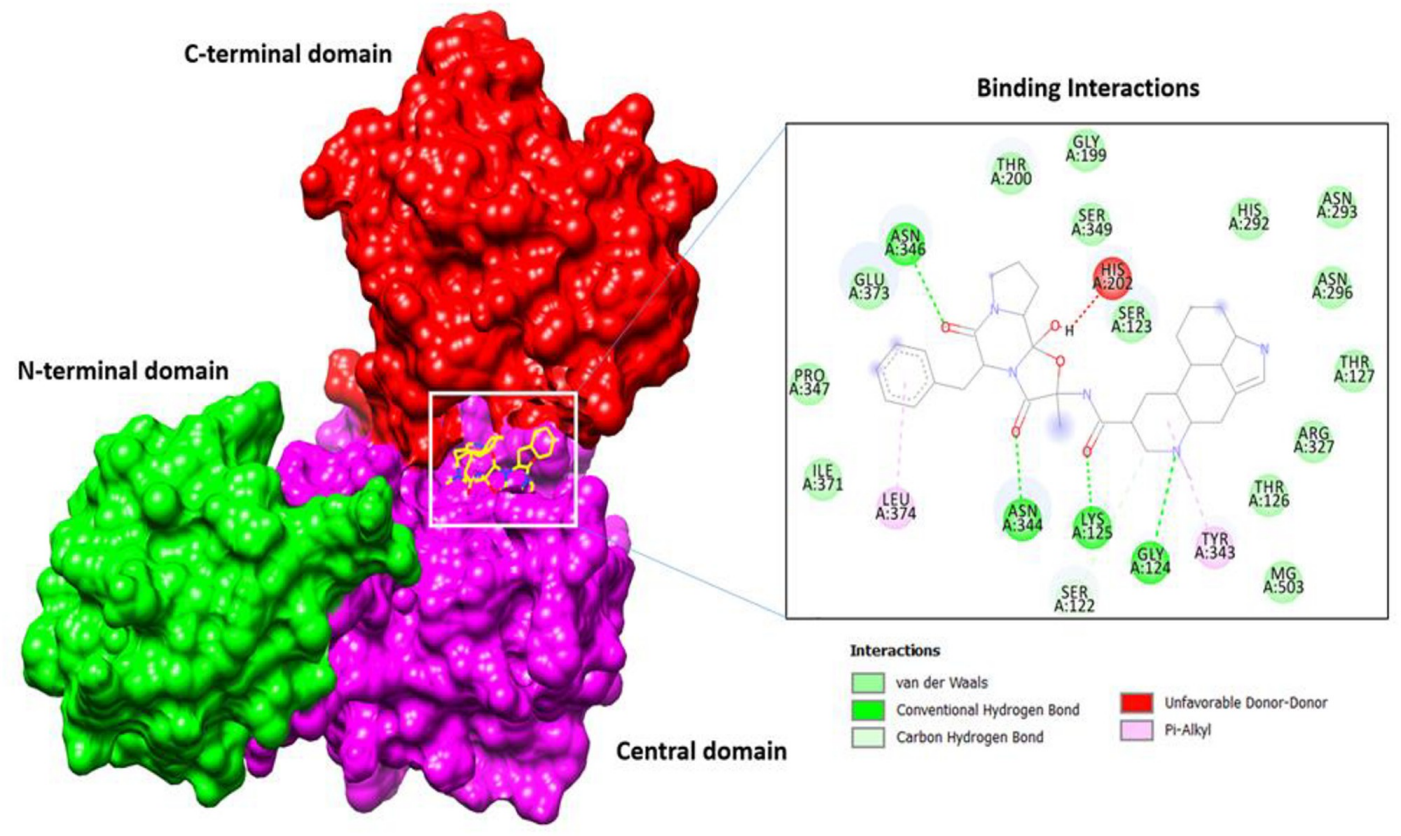
Binding mode of the compound (shown in yellow stick) and residues from ATP binding cavity from both interacting domains central (Magenta surface) and C-terminal (red surface) are shown in closed view.

**Fig 3:**
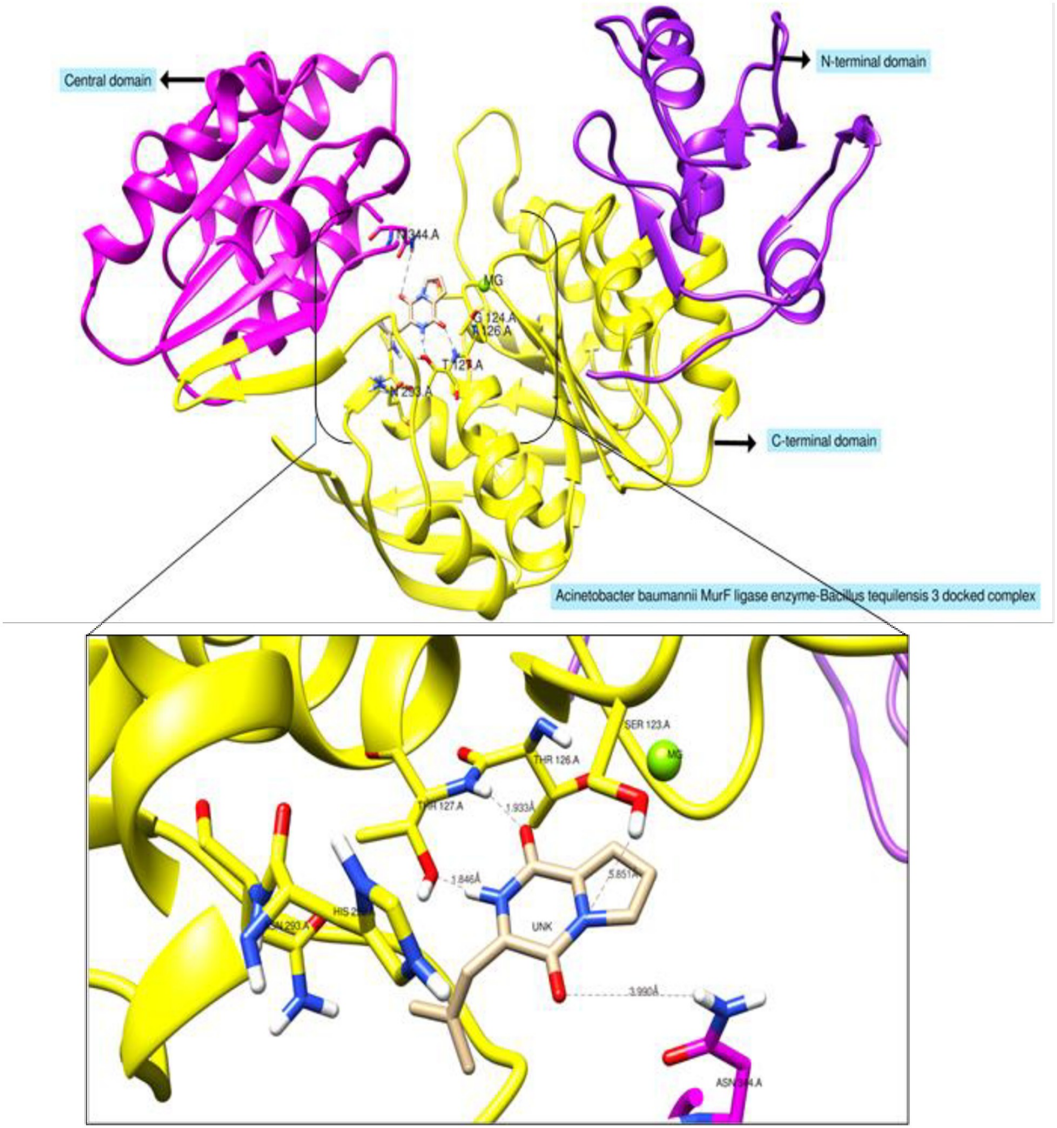
Docked complex of *Bacillus tequilensis* 3 (3-isobutyl hexahydropyrrolo[1,2-a] pyrazine-1,4-dione) compound with MurF ligase enzyme from *Acinetobacter baumannii* and their binding interaction at ATP binding cavity of between central and C-terminal domain with conventional hydrogen bond.

Most of the compounds showed high affinity for the MurF ligase enzyme (The Mur ligases (MurC, MurD, MurE and MurF) enzymes play an essential role in the biosynthesis of peptidoglycan of both Gram-negative and Gram-positive bacterial cell-wall and thus represent attractive targets for the design of novel antibacterial drugs) among the tested receptors (Table 3). Out of which Compound *Stenotrophomonas_maltophilia*_11 for instance was ranked as an excellent binder of the MurF ligase enzyme by showing stable conformer at ATP binding cavity between C-terminal and Central domain. Relative to the control ATP molecule, the said compound seems major and stable affinity by requiring binding energy of -10.2 kcal/mol with “rmsd/ub and rmsd/Ib” equal to 0. The binding mode and chemical interactions at the docked position are presented in (Fig 2). The 7-methyl 2,3,3a,3a^1^,6,6a,7,8,9,10,10a,10b-dodecahydro-1H-4λ^2^-indolo[4,3-*f*g] quinoline-9-carboxamide fragment of the compound *S*. *maltophilia*_11 tilted more towards the central domain where it can be seen in majority of hydrophobic interactions. Opposite to this, the N-(5-benzyl-10b-hydroxy-2-methyl-3, 6-dioxooctahydro-2H-oxazolo [3, 2-a] pyrrolo [2, 1-c] pyrazin-2-yl) form amide favors interactions with the C-terminal domain through balanced interactions of hydrophobic and hydrophilic. Additionally, we also visualized biological potent *B*. *tequilensis* 3 that favors binding to the ATP binding domain with strong network of hydrogen bonding and rich pattern of hydrophobic interactions (Fig 3).

**Table 3:** Docking scores of the compounds with respect MurF receptor.

Ligand	Binding Affinity	rmsd/ub	rmsd/lb
*Stenotrophomonas_maltophilia_11*	-10.2	0	0
*Bacillus_tequilensis_7*	-9.7	3.207	2.269
*Enterobacter_cloacae_7*	-9.5	7.526	4.775
*Enterobacter_cloacae_8*	-9.2	8.089	5
*Bacillus_tequilensis_5*	-8.7	14.601	11.879
*Erwinia_sp_4*	-8.5	0	0
*Stenotrophomonas_maltophilia_9*	-8.4	25.915	22.03
*Bacillus_tequilensis_6*	-8.4	25.444	21.241
*Bacillus_subtilis_1*	-8.3	29.172	26.288
*Bacillus_subtilis_2*	-8.3	23.649	21.272
*Bacillus_subtilis_3*	-8.1	0	0
*Bacillus_tequilensis_3*	-8	0	0
*Enterobacter_hormaechei_1*	-7.9	35.942	33.695
*Erwinia_sp_1*	-7.6	5.269	2.804
*Bacillus_tequilensis_1*	-7.6	4.959	2.11
*Enterobacter_hormaechei_2*	-7.5	0	0
*Enterobacter_cloacae_6*	-7.4	5.637	2.353
*Enterobacter_cloacae__6*	-7.4	5.512	2.883
*Bacillus_subtilis_4*	-7.4	24.513	19.326
*Enterobacter_cloacae_1*	-7.3	0	0
*Enterobacter_cloacae_3*	-7.3	0	0
*Stenotrophomonas_maltophilia_5*	-7.2	2.761	1.35
*Bacillus_tequilensis_4*	-7.2	5.887	2.227
*Enterobacter_hormaechei_4*.	-7.2	22.75	17.907
*Stenotrophomonas_maltophilia_4*	-7.1	0	0
*Stenotrophomonas_maltophilia_6*	-7.1	3.502	2.05
*Pantoea dispersa 1*	-7.1	28.219	25.052
*Bacillus_tequilensis_2*	-7.1	8.05	4.379
*Stenotrophomonas_maltophilia_7*	-6.9	5.771	2.243
*Erwinia_sp_3*	-6.9	4.646	2.499
*Enterobacter_cloacae_2*	-6.9	26.003	23.02
*Enterobacter_hormaechei_4*	-6.9	28.918	25.597
*Stenotrophomonas_maltophilia_10*	-6.9	16.267	14.352
*Enterobacter_hormaechei_6*	-6.9	23.835	22.075
*Stenotrophomonas_maltophilia_3*	-6.8	0	0
*Enterobacter_hormaechei_3*	-6.8	5.644	2.305
*Stenotrophomonas_maltophilia_2*	-6.8	15.387	10.66
*Enterobacter_hormaechei_5*.	-6.8	2.568	1.518
*Stenotrophomonas_maltophilia_1*	-6.8	6.175	2.664
*Stenotrophomonas_maltophilia_8*	-6.7	0	0
*Enterobacter_hormaechei_5*	-6.7	14.433	13.068
*Erwinia_sp_2*	-6.7	8.347	2.399
*Pantoea _cypripedii_5*	-5	0	0
*Pantoea_cypripedii_7*	-4.9	10.525	2.102
*Pantoea _cypripedii_3*	-4.9	10.545	2.004
*Pantoea _cypripedii_4*	-4.8	8.334	4.892
*Pantoea _cypripedii _1*	-4.7	10.997	2.686
*Pantoea _cypripedii _6*	-4.5	10.25	1.73
*Pantoea _cypripedii _2*	-4.5	12.189	3.422
*Pantoea _dispersa _3*	-4.5	10.664	2.827
*Pantoea _dispersa _2*	-4.5	27.193	22.703
Control	-8.9	0	0

#### Molecular dynamics simulations

**Fig 4:**
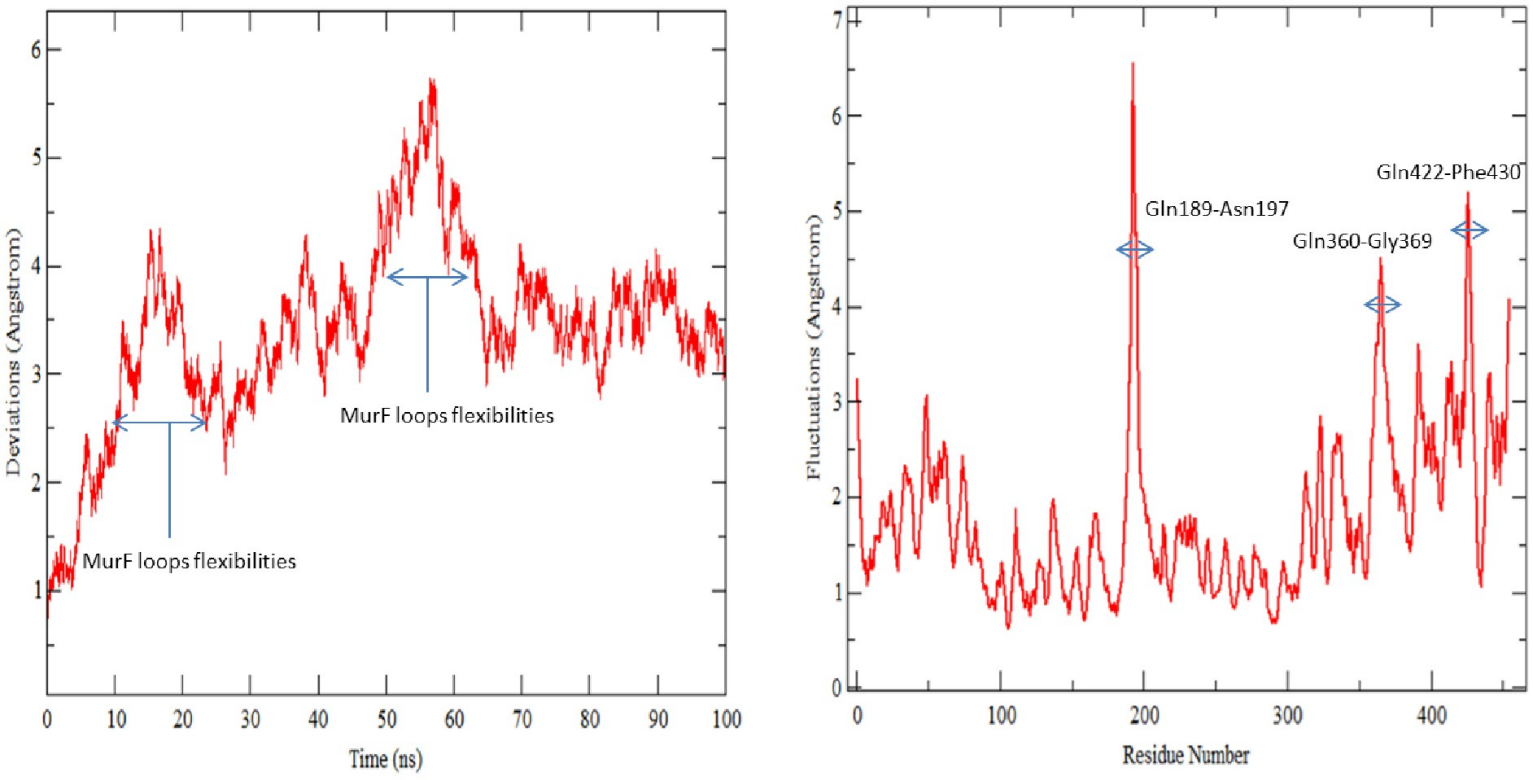
Rmsd (left) and rmsf (right) plots for MurF enzyme and compound complex.

Molecular dynamics simulations were further applied on the enzyme-inhibitor complex to gain vital information about the system dynamics and affirm the stability of the compound confirmation at the docked site. Two structural assays were performed on the simulation trajectories: root mean square deviations (rmsd) and root mean square fluctuations (rmsf) as shown in (Fig 4). The stability of the system was evaluated first by calculating rmsd for all frames generated by the dynamic assay to measure the mean displacement change of all atoms from their original reference position predicted by docking. The mean rmsd of the system is 3.39 Å (maximum, 5.7 Å), a reflection of the good stability of the receptor structure. Majority of the deviations in the protein structure can be observed in first phase of simulation i.e., initial 50 ns, followed by a stable trend of rmsd plot till the end. The first phase deviations were investigated as loop flexibility making the protein relax to adopt proper changes needed for proper hold of the compound.

Towards the end period of simulation, the protein achieved greater stability in the presence of the compound. Throughout the simulated period, the compound retained its original binding mode predicted by docking and govern by enriching hydrophilic and hydrophobic interactions. Further, protein residues stability in the presence of the compound was unraveled by performing rmsf assay. The mean rmsf of the system is 1.75 Å depicting good affinity and stable nature of the protein residues in the presence of the compound. Few fluctuations can be seen in the rmsf plot i.e., Gln189-Asn197, Gln360-Gly369, and Gln422-Phe430. These regions are present in the central and C-terminal domain, both said domains are in direct contact with the compounds and these fluctuations seem to be adoptive changes required for proper recognition and stronghold on the compound.

#### Estimation of binding free energies

The binding free energies between the compound and MurF enzyme revealed by both MMPBSA and MMGBSA methods are summarized in (Table 4). As can be notice from the table, the system energy is quite favorable resulting in the formation of a highly stable complex. Both approaches identified gas-phase energy very decreased (-102.3795 kcal/mol) with balanced contribution from both van der Waals and electrostatic energy. This indicates both hydrophobic and hydrophilic chemical interactions are playing an eloquent role in the stability of the compound at the docked site. Further, it emphasizes on the less role of water molecules in interactions between the compound and enzyme. The latter statement can be supported by the fact that solvation energy contribution is non-favorable (82.6597 kcal/mol in MMGBSA and79.9093 kcal/molin MMPBSA). The net binding energy for the system is -19.7198 kcal/mol in MMGBSA and -22.4702 kcal/mol in MMPBSA. Both these values are in full agreement of the good and stable affinity of the compound for the enzyme.

**Table 4:** Binding free energies reported by MMGBSA and MMPBSA methods for the MurF-compound complex.

GENERALIZED BORN			
Energy Component	Average	Std. Deviations	Std. Error of Mean
**VDWAALS**	-32.8323	3.5645	0.3565
**EEL**	-69.5472	9.1718	0.9172
**EGB**	87.3292	8.3345	0.8334
**ESURF**	-4.6695	0.4486	0.0449
**DELTA G gas**	-102.3795	8.9640	0.8964
**DELTA G solv**	82.6597	8.4228	0.8423
**DELTA TOTAL**	-19.7198	3.8138	0.3814
**POISSON BOLTZMANN**			
**VDWAALS**	-32.8323	3.5645	0.3565
**EEL**	-69.5472	9.1718	0.9172
**EPB**	83.7909	8.4186	0.8419
**ENPOLAR**	-3.8815	0.2340	0.0234
**EDI+**	0.0000	0.0000	0.0000
**SPER**			
**DELTA G gas**	-102.3795	8.9640	0.8964
**DELTA G solv**	79.9093	8.4342	0.8434
**DELTA TOTAL**	-22.4702	3.4860	0.3486

## Discussion

According with the previous reported literature, our study showed the isolation of endophytic (Bacteria) from medicinally important plant *F*. *indica* and the biological screening of their crude extracts to assess their biological potential. Medicinal plants are rich sources of secondary metabolites, and the endophytes of these plants spend their whole life cycle within their tissues without causing any signs of disease or infections [21]. Endophytes are a cohort of microbes that could generate a plethora of novel and highly potent compounds of pharmaceutical and agricultural importance [22]. Search for new antimicrobial agents is the need of time as pathogenic strains are developing resistance to the commonly used antibiotics. Endophytic bacteria secreted secondary metabolites production may be mimicked in *In-vitro* conditions for therapeutic applications [23]. The ESKAPE such as (*Enterococcus faecium*, *Staphylococcus aureus*, *Klebsiella pneumoniae*, *Acinetobacter baumannii*, *Pseudomonas aeruginosa*, and *Enterobacter* species) bacterial pathogens are a group of commonly MDR (Multiple drug resistance) organisms that arise the antibiotic resistance problem. Among this group, *A*. *baumannii* is a naturally competent organism maintaining and acquiring multiple genetic elements encoding antimicrobial resistance determinants [24].

Keeping the same phenomenon in mind, in the present study we have screened the metabolites to determine their antibacterial potency against multi drug-resistant strains (MDRs) of pathogenic bacteria. Results demonstrated that extracts revealed antagonistic potential against all pathogenic strains. Among the endophytic bacterial Extract of S. maltophilia shows the highest zone of inhibition as 15.11±0.11mm and 11.3±0.16 against *S*.*caseolyticus* and *A*.*baumanni* with 33.3 and 50μg/mL MIC respectively. These results agree with a previous study where the antimicrobial potential of endophytic species was evaluated against human pathogenic microbes [25].

Inhibitory power of endophytic bacterial strains could be observed from the formation of clear zones around their isolate’s colonies [26]. The clear zones indicate the occurrence of antimicrobial metabolites, secreted by endophytic strains that inhibit or kill the pathogenic bacteria. The degree of bacterial susceptibility to antibacterial substances or antimicrobial potential of a pure compound could be tested through this inhibitory test. Suggested inhibitory mechanisms of endophytic strains could be cell wall synthesis inhibition, bacterial metabolism disruption, nucleic acid or protein synthesis inhibition, or alteration in cell membrane permeability [27]. The tested extracts successfully inhibited both gram-positive and gram-negative pathogenic bacterial strains. This phenomenon is considered as broad-spectrum antibacterial potential [28]. Based on the current findings we conclude that endophytic bacterial strains isolated from *Fagonia indica* exhibit a broad-spectrum antibacterial potential against human pathogenic bacterial strains.

To confirm the presence of compounds that are responsible for pharmacological activities the obtained extracts were further subjected to chemical analysis by the GC-MS method. There is a growing awareness in correlating the phytochemical compounds and their biological activities. Gas chromatogram Mass spectrometry (GC-MS) explored Methyl 2-(2-((2, 6 dichlorophenyl) amino) phenyl) acetate (Diclofenac Methyl Ester) with molecular formula C_15_H_13_C_l2_NO_2_ having analgesic and anti-inflammatory properties. 2-methyl hexadecan-1-ol is recorded to have insecticidal, anti-tuberculosis, anti-inflammatory, antioxidant and antimicrobial activities [29]. n-Hexadecanoic acid and Pentadecanoic acid on the other hand which is commonly known as Palmitic acid and Pentadecylic acid is the ester fatty acids with pesticide, nematicide, lubricant, anti-androgenic, antibacterial, antioxidant, hypo-cholesterolemic and hemolytic 5-alpha reductase inhibition properties [30].

### Proposed mechanism for antibacterial activity

**Fig 5:**
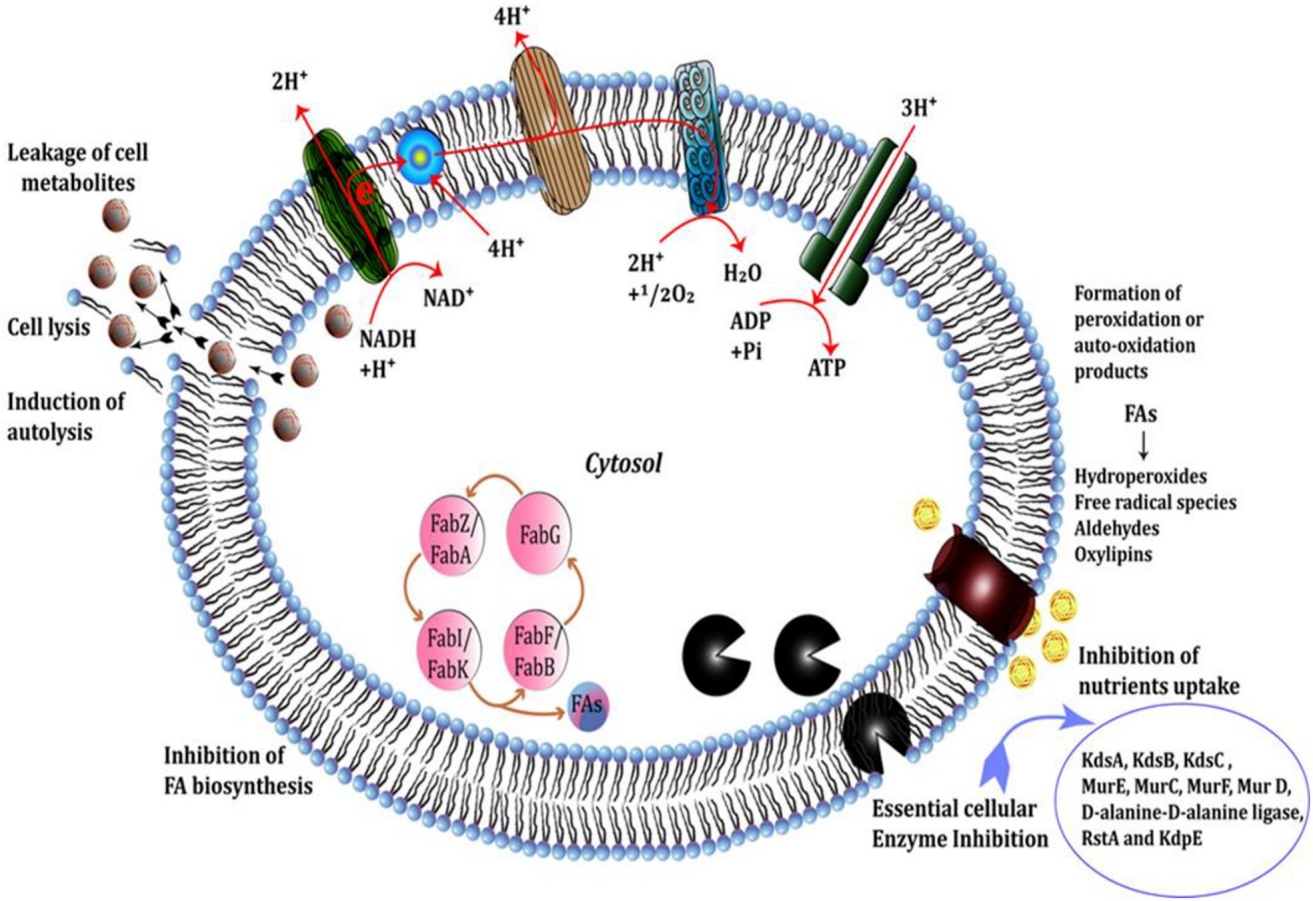
Schematic representation of cell targets and mechanisms of antibacterial activity of fatty acids.

Antibacterial lipids, amide, glycerol, aldehyde, esters are biologically active constituents, generally synthesized by living organisms for their life processing. Further, these compounds have been reported as bactericidal agents. The antibacterial activity of such compounds depends upon their structural characteristic, target sites and mechanism of interactions. (Fig 5) shows proposed antibacterial mechanisms based on characterised extracts (i.e., GC-MS analysis). While focusing on the antibacterial activity the interaction of antimicrobial lipid such as fatty acids (the major constituent of our compounds) with bacterial cell membranes can increase membrane permeability (generates variable size pores). Increased permeability leads to membrane destabilization, leakage of cytosolic contents, complete cell lysis, autolysis and an increase in K+ efflux and eventually causes the death of the bacterium. Another mechanism is to disrupt in Electron Transport chain (ETC) and oxidative phosphorylation reaction both of which produced ATP as a source of energy via ATP synthase in case of oxidative phosphorylation reaction. When one of the steps in the electron transport chain and oxidative phosphorylation is interrupted, this result in a reduction of ATP production an essential source of energy due to which the bacterium becomes deprived and leads to eventually cell death.

Another significant mechanism of antimicrobial lipid (FAs) that affect bacterial cells is inhibiting the activity of membrane-associated proteins/ enzymes including KdsA, KdsB, KdsC, MurE, MurC, MurF, MurD (Mur ligases catalyze the formation of a peptide or amide bond between the UDP-substrate and the condensing amino acid), D-alanine-D-alanine ligase, RstA and KdpE because all of these are ATP-dependent. It can be concluded that there are multiple mechanistic approaches by which FAs might inhibit bacterial growth.

After representation of bacteria cell targets mechanisms of the biologically active constituents present in the compounds identified with the help of GC-MC, these compounds were further used for molecular docking studies to determine the affinity of these compounds against different broad-spectrum receptor proteins of the bacterial cell.

Traditional medicinal knowledge has also given clues to the discovery of valuable drugs, despite the advantages of screening techniques [31]. There is a growing awareness in correlating the phytochemical compounds with their biological activities [32]. Computational studies of the extracted compounds were additionally performed to provide combined experimental and in silico data to the readers for possible binding targets in a bacterial cell. A rigorous antibacterial target screening was done where the compounds unveil to show favorable binding to the bacterial cell proteins. Dynamically the compounds exhibit stability behavior with the MurF protein, a peptidoglycan biosynthesis enzyme. The docking simulation predictions were affirmed by widely acceptable MM-PBSA binding free energy that quantifies the domination of van der Waals and electrostatic interactions in binding. This set of information holds significant importance for the future of optimization of these lead compounds to produce highly potent derivatives.

## Conclusion

From the preliminary study, it can be concluded that endophytic bacteria are a dependable source of bioactive compounds having the potentials to be a promising source of significant antibacterial efficacy. Where *S*. *maltophilia* represents remarkable antibacterial potency toward *S*.*caseolyticus* and *A*.*baumanni* with 15.2±0.11 mm and 11.3±0.16mm zones of inhibitions having 33.3,50μg/ml MIC respectively. GC-MS result of the bacterial crude revealed the identification of fifty different compounds having important constituents responsible for antibacterial activity. Molecular docking result revealed that the compounds show affinity and holds a promising lead target toward the broad-spectrum receptor proteins involved in the biosynthesis of pathogenic bacterial cells outer membrane, among which the Compound N-(5-benzyl-10b-hydroxy-2-methyl-3,6-dioxooctahydro-8H-oxazolo[3,2-α]pyrrolo[2,1 c] pyrazin-2-yl)-7-Methyl -2,3, 3a,3a^1^,6,6a,7,8,9,10,10a,10b-dodecahydro-1H-4λ^2^-indolo[4,3-*f*g]quinoline-9-carboxamide for instance was ranked as an excellent binder of the MurF enzyme with binding energy -10.2 kcal/mol (rmsd/ub and rmsd/Ib equal to zero) and could be a potent antibacterial compound. Moreover, it is important to draw the attention of the scientific community towards this field of research for the exploration of more valuable compounds from endophytic bacteria having therapeutic potential.

## Supporting information

S1 Fig Phylogenetic tree of isolated bacterial partial 16S rRNA sequences along with the sequences from selected references strains. The analysis was conducted with Bioedit and MEGA 07 using neighbor-joining method (Bootstrap analysis with 500 replicates).
(DOCX)

S2 Fig Antibacterial assay of secondary metabolites against *Staphylococcus epidermis*, *Staphylococcus aureus*, *Staphylococcus caseolyticus*, Methicillin resistance *Staphylococcus aureus*, *Enteriobactor cloacae* and *Acinetobacter baumannii*. (**P.C**) represent the positive control (Ampicillin, Meropenem) while (**N.C**) represent the negative control (DMSO) zones of inhibition.
(DOCX)

S3 Fig Molecular ion and daughter ion peaks as obtained through GC-MS. (DOCX)

S1 Table Different compounds obtained from gas chromatography-mass spectrometry (GC-MS) analysis of bacterial crude extracts with their retention time (RT), molecular weight (MW) molecular formula (MF) and concentration (peak area %). (DOCX)

S1 Graphical abstract (TIF)
