# Supplementary material for: Endophytic bacteria of Fagonia indica Burm. f revealed to harbour rich secondary antibacterial metabolites
Source: PLoS One. 2022 Dec 15;17(12):e0277825. doi: 10.1371/journal.pone.0277825 (PMC9754247; doi:10.1371/journal.pone.0277825)
Supplement: S1 Fig — The analysis was conducted with Bioedit and MEGA 07 using neighbor-joining method (Bootstrap analysis with 500 replicates). (DOCX) [file pone.0277825.s001.docx]

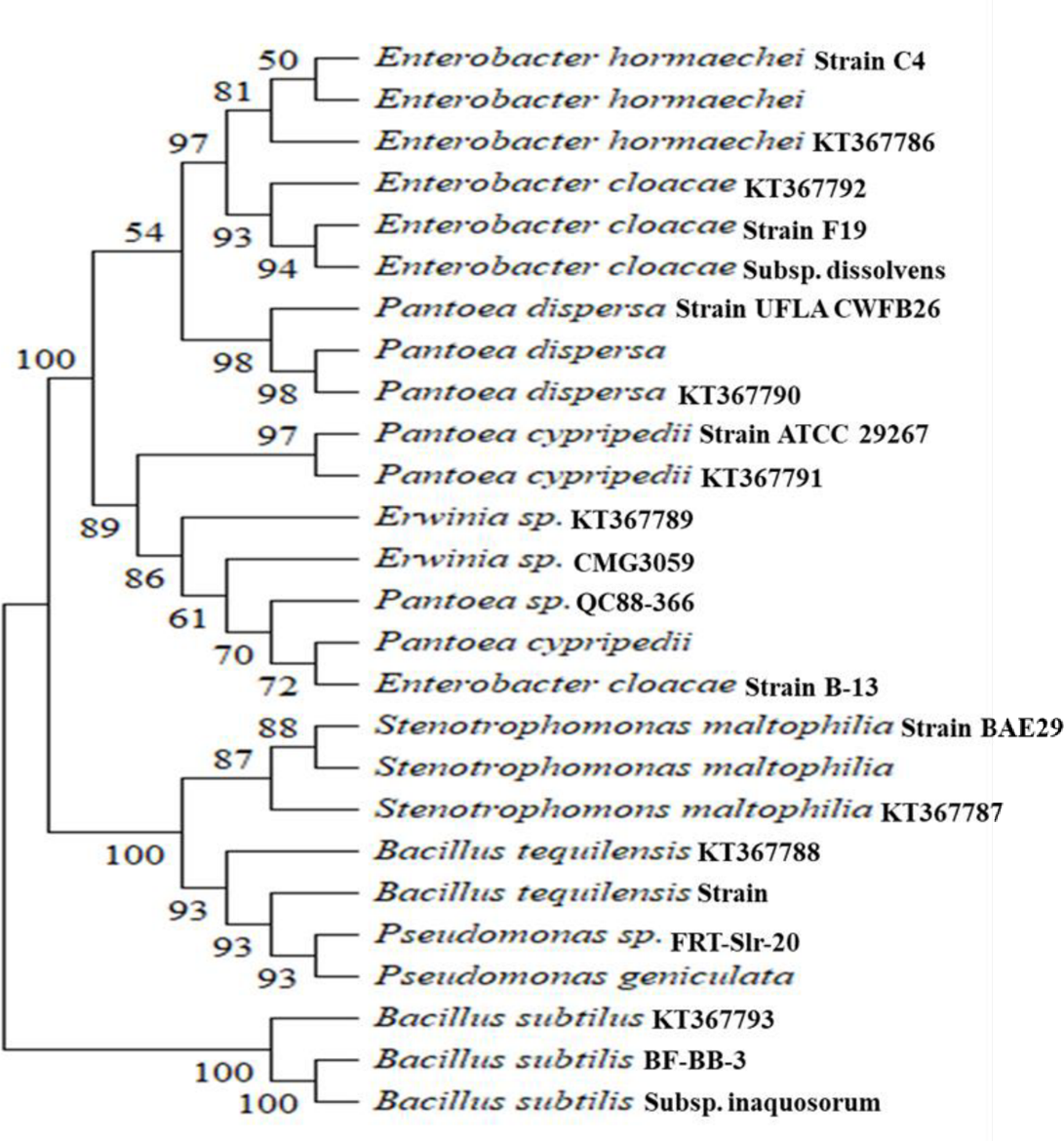


**S1 Fig.** Phylogenetic tree of isolated bacterial partial 16S rRNA sequences along with the sequences from selected references strains. The analysis was conducted with Bioedit and MEGA 07 using neighbor-joining method (Bootstrap analysis with 500 replicates)
