# Supplementary material for: Endophytic bacteria of Fagonia indica Burm. f revealed to harbour rich secondary antibacterial metabolites
Source: PLoS One. 2022 Dec 15;17(12):e0277825. doi: 10.1371/journal.pone.0277825 (PMC9754247; doi:10.1371/journal.pone.0277825)
Supplement: S2 Fig — (P.C) represent the positive control (Ampicillin, Meropenem) while (N.C) represent the negative control (DMSO) zones of inhibition. (DOCX) [file pone.0277825.s002.docx]

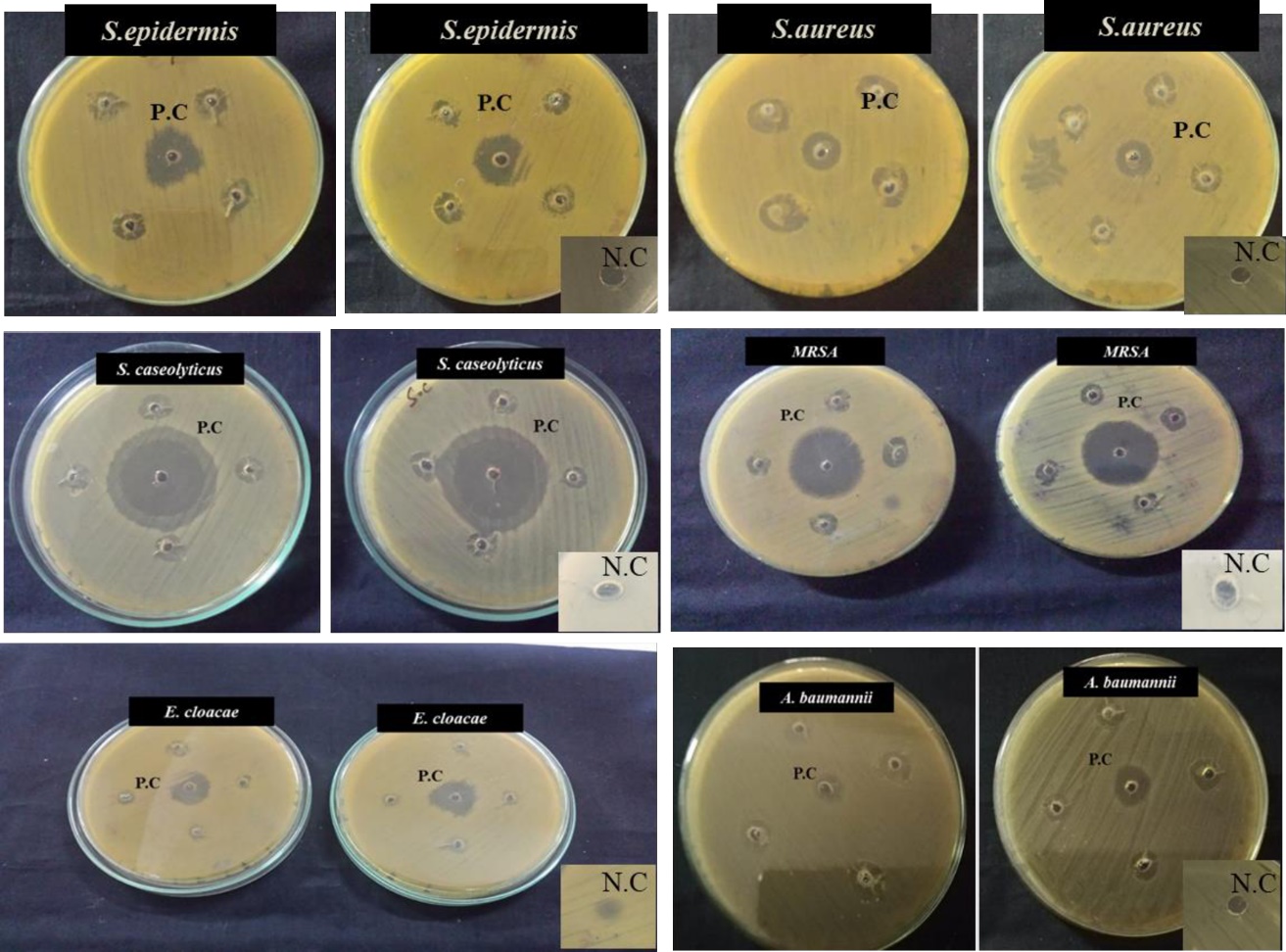


**S2 Fig.** Antibacterial assay of secondary metabolites against *Staphylococcus epidermis*, *Staphylococcus aureus*, *Staphylococcus caseolyticus*, Methicillin resistance *Staphylococcus aureus*, *Enteriobactor cloacae* and *Acinetobacter baumannii*. (**P.C**) represent the positive control (Ampicillin, Meropenem) while (**N.C**) represent the negative control (DMSO) zones of inhibition
