# Supplementary material for: Endophytic bacteria of Fagonia indica Burm. f revealed to harbour rich secondary antibacterial metabolites
Source: PLoS One. 2022 Dec 15;17(12):e0277825. doi: 10.1371/journal.pone.0277825 (PMC9754247; doi:10.1371/journal.pone.0277825)
Supplement: S3 Fig — (DOCX) [file pone.0277825.s003.docx]

N-(5-benzyl-10b-hydroxy-2-methyl-3,6-dioxooctahydro-8H-oxazolo[3,2-α] pyrrolo [2,1 c] pyrazin-2-yl)-7-methyl 2,3,3a,3a^1^,6,6a,7,8,9,10,10a,10b-dodecahydro-1H-4λ^2^-indolo[4,3-*f*g] quinoline-9-carboxamide

Ethylbenzene

3-isobutylhexahydropyrrolo[1,2-a] pyrazine-1,4-dione


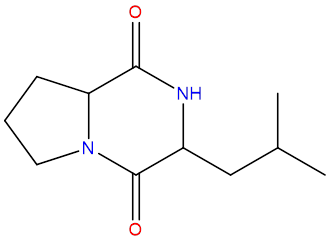

(Z)-16-((Z)-2-hydroxy-1-(methoxyimino) ethyl)-10,13,17-trimethyl-4,15,16,17-tetrahydro-3H-10l5,13l5-cyclopenta[a]phenanthren-3-one O-methyl oxime

8,8-dimethyl-3,3a,4,5,6,7,8,8b octahydro-2H-indeno[1,2-b] furan-2-one

N-(6-methoxy-2-methyl-8-((trimethylsilyl)oxy) hexahydropyrano[3,2-d] [1,3,2] dioxaborinin-7-yl) acetamide

2-(2-chloro-4-methylphenoxy)-N, N-diethylethan-1-amine

(1-ethyl-4-methyl-1,1a,2,3,4,4a,9,10 octahydrocyclopropa [3',4’] pyrido [2',3':3,4] cyclopenta [1,2-b] indol-10-yl) methyl acetate

N'-(3,6-dichloro-2,7-bis(2-(diethylamino) ethoxy)-9H-fluoren-9 ylidene) pivalohydrazide

3-isobutylhexahydropyrrolo[1,2-a] pyrazine-1,4-dione

**S3 Fig.** Molecular ion and daughter ion peaks as obtained through GC-MS
