# Supplementary material for: Endophytic bacteria of Fagonia indica Burm. f revealed to harbour rich secondary antibacterial metabolites
Source: PLoS One. 2022 Dec 15;17(12):e0277825. doi: 10.1371/journal.pone.0277825 (PMC9754247; doi:10.1371/journal.pone.0277825)
Supplement: S1 Table — (DOCX) [file pone.0277825.s004.docx]

**S1 Table.** Different compounds obtained from Gas Chromatography-Mass Spectrometry (GC-MS) analysis of bacterial crude extracts with their retention time (RT), Molecular weight (MW) Molecular formula (MF) and concentration (peak area %)

| **Endophytic Bacteria** | | | | | | | | | |
| --- | --- | --- | --- | --- | --- | --- | --- | --- | --- |
| ***Enterobacter hormaechei*** | | | | | | | | | |
| Peak | | Retention Time | IUPAC Name | Chemical Structure | Molecular Formula and functional groups | | | Formula  weight | % Composition  Area |
| 1 | | 22.70 | Methyl 2-(2-((2,6 dichlorophenyl) amino) phenyl) acetate |  | C_15_H_13_C_l2_NO_2_  Ester | | | 310.17 g/mol | 18% |
| 2 | | 23.21 | Bis (6-methylheptyl) phthalate |  | C_24_H_38_O_4_  Ester of anhydride | | | 390.56 g/mol | 45% |
| 3 | | 23.93 | (E)-octadec-5-enal |  | C_18_H_34_O  Aldehyde | | | 266.5 g/mol | 26% |
| 4 | | 25.09 | 1,1-bis(dodecyloxy)hexadecane |  | C_40_H_82_O_2_  Ether linkage | | | 595.09 g/mol | 30% |
| 5 | | 29.67 | (2-methylhexadecyl) holmium |  | C_17_H_35_HO  Alcohol | | | 404.40g/mol | 54% |
| 6 | | 32.30 | (E)-hexadec-4-enal |  | ‎C_16_H_30_O  Aldehyde  ‎ | | | 238.82g/mol | 56% |
|  | | | ***Stenotrophomons maltophilia*** | | | | | |  |
| 1 | 3.92 | | p-xylene |  | C_6_H_4_(CH_3_)_2_  Aromatic hydrocarbon | | 106.16 g/mol | | 87.3% |
| 2 | 4.17 | | Ethylbenzene |  | C_8_H_10_  Aromatic hydrocarbon | | 106.16 g/mol | | 63.4% |
| 3 | 8.99 | | 3.Eicosene (E)- |  | C_20_H_40_  Alkene | | 280.53 g/mol | | 40% |
| 4 | 10.01 | | Pentadecanoic acid |  | C_15_H_30_O_2_  Fatty acid | | 242.3975 g/mol | | 44.4% |
| 5 | 10.63 | | 2-methoxy-13-methylpentadec-1-ene |  | C_17_H_34_O  Alkene | | 254.46g/mol | | 25% |
| 6 | 11.03 | | n-Hexadecanoic acid |  | C_16_H_32_O  Fatty acid | | 256.43 g/mol | | 36% |
| 7 | 11.65 | | N-(2-hydroxyethyl) decanamide |  | C_12_H_25_NO_2_  Amide | | 215.34 g/mol | | 32% |
| 8 | 11.92 | | 2-methylhexadecan-1-ol |  | C_17_H_36_O  Alcohol | | 256.48 g/mol | | 45.4% |
| 9 | 12.35 | | 3a-methyl hexadecahydro cyclopenta[b]fluoren-3-ol |  | [C_17_H_28_O](https://pubchem.ncbi.nlm.nih.gov/#query=C7H12O)  Cyclic alcohol | | 248.41g/mol | | 51.3% |
| 10 | 12.86 | | 2,3-bis(heptadec-1-en-2-yloxy) propan-1-ol |  | C_37_H_72_O_3_  Alcohol | | 564.98 g/mol | | 20% |
| 11 | 15.38 | | N-(5-benzyl-10b-hydroxy-2-methyl-3,6-dioxooctahydro-8H-oxazolo[3,2-α] pyrrolo [2,1 c]pyrazin-2-yl)-7-methyl 2,3,3a,3a^1^,6,6a,7,8,9,10,10a,10b-dodecahydro-1H-4λ^2^-indolo[4,3-*f*g]quinoline-9-carboxamide |  | C_33_H_35_N_5_O_5_  Amide | | 588.66 g/mol | | 12.6% |
|  | | | ***Bacillus tequilensis*** | | | | | |  |
| 1 | 8.84 | | (2-phenyl-1,3-dioxolan-4-yl) methyl (E) octadec-9-enoate |  | C_28_H_44_O_4_  Ester | | 444.6 g/mol | | 16.6% |
| 2 | 10.80 | | 2,2,8,8-tetramethyl-3,7-dioxa-2,8-disilanonan-5-yl (9E,12E,15E)-octadeca-9,12,15-trienoate |  | C_27_H_52_O_4_Si_2_  Ester | | 496.9 g/mol | | 18.1% |
| 3 | 11.38 | | 3-isobutylhexahydropyrrolo[1,2-a] pyrazine-1,4-dione | 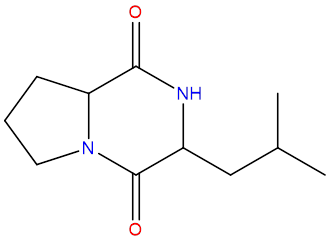 | ‎ C_11_H_18_N_2_O_2_  ketone | | 210.27 g/mol | | 49.4% |
| 4 | 11.95 | | heptadec-1-en-2-ol |  | C_17_H_34_O  Alcohol | | 254.46g/mol | | 48.3% |
| 5 | 12.47 | | 3a methylhexa decahydrocyclopenta[a]fluoren-3-ol |  | C_17_H_28_O  Cyclic alcohol | | 248.41 g/mol | | 86% |
| 6 | 14.38 | | (Z)-N'-(3,6-dichloro-2,7-bis(2-(diethylamino) ethoxy)-1-methyl-1,2,3,4,4a,9a-hexahydro-9H-fluoren-9-ylidene) pivalohydrazide |  | [C_31_H_50_Cl_2_N_4_O_3_](https://pubchem.ncbi.nlm.nih.gov/#query=C30H42Cl2N4O3)  Hydrazine | | 597.6 g/mol | | 30.7% |
| 7 | 15.38 | | (E)-17-hydroxy-17-((E)-2-hydroxy-1-(methoxyimino) ethyl)-10,13-dimethyl 1,2,6,7,8,9,10,11,12,13,14,15,16,17-tetradecahydro-3H-cyclopenta[a]phenanthren-3-one O-methyl oxime |  | [C_23_H_36_N_2_O_4_](https://pubchem.ncbi.nlm.nih.gov/#query=C22H33NO3)  Imine | | 404.5 g/mol | | 64% |
|  | | | ***Erwinia spp*** | | | | | |  |
| 1 | 21.99 | | 2,4-di-tert-butylphenol |  | C_14_H_22_O  Cyclic alcohol | | 206.32 g/mol | | 25.9% |
| 2 | 22.65 | | (E)-octadec-5-enal |  | [C_18_H_34_O](https://pubchem.ncbi.nlm.nih.gov/#query=C18H34O)  Aldehyde | | 266.5 g/mol | | 47.3% |
| 3 | 23.87 | | 1,1-bis(dodecyloxy)hexadecane |  | [C_40_H_82_O_2_](https://pubchem.ncbi.nlm.nih.gov/#query=C40H82O2)  Alkane | | 595.1 g/mol | | 23% |
| 4 | 32.28 | | N'-(3,6-dichloro-2,7-bis(2-(diethylamino) ethoxy)-9H-fluoren-9-ylidene) pivalohydrazide |  | [C_30_H_42_Cl_2_N_4_O_3_](https://pubchem.ncbi.nlm.nih.gov/#query=C30H42Cl2N4O3)  Hydrazine | | 577.6 g/mol | | 74% |
|  | | | ***Pantoea Dispersa*** | | | | | |  |
| 1 | 8.24 | | Dichloromethane |  | CH_2_Cl_2_  Haloalkane | | 84.93 g/mol | | 39.7% |
| 2 | 9.22 | | Methylsulfonylmethane |  | (CH₃)₂SO₂  Organosulfur | | 94.13 g/mol | | 60.2% |
| 3 | 12.36 | | (1-ethyl-4-methyl-1,1a,2,3,4,4a,9,10-octahydrocyclopropa [3',4’] pyrido[2',3':3,4]cyclopenta[1,2-b]indol-10-yl)methyl acetate |  | [C_21_H_26_N_2_O_2_](https://pubchem.ncbi.nlm.nih.gov/#query=C18H20N2O3)  Ester | | 338.45g/mol | | 73.6% |
|  | | | ***Pantoea cypripedii*** | | | | | |  |
| 1 | 9.11 | | 8,8-dimethyl-3,3a,4,5,6,7,8,8b octahydro-2H-indeno[1,2-b] furan-2-one |  | C_13_H_18_O_2_  ketone | | 206.28 g/mol | | 22% |
| 2 | 10.76 | | Pentadecanoic acid |  | C_15_H_30_O_2_  Fatty acid | | 242.3975 g/mol | | 47.1% |
| 3 | 11.70 | | 2,3-bis(heptadec-1-en-2-yloxy) propan-1-ol |  | C_37_H_72_O_3_  alcohol | | 564.98 g/mol | | 52.6% |
| 4 | 12.00 | | 1,1-bis(dodecyloxy)hexadecane |  | C_40_H_82_O_2_  Alkane derivative | | 595.1 g/mo | | 59.3% |
| 5 | 14.16 | | (Z)-prop-1-ene-1,2,3-triyl tridocosanoate |  | [C_69_H_132_O_6_](https://pubchem.ncbi.nlm.nih.gov/#query=C6H12N6O3)  Ester | | 1057.81g/mol | | 41.9% |
| 6 | 14.39 | | 2-((2-chlorocyclohexyl) oxy)-N,N-diethylethan-1-amine |  | [C_12_H_24_ClN](https://pubchem.ncbi.nlm.nih.gov/#query=C6H12ClN)  Amine | | 233.78g/mol | | 62.5% |
| 7 | 15.45 | | N-(6-methoxy-2-methyl-8-((trimethylsilyl)oxy) hexahydropyrano[3,2-d][1,3,2]dioxaborinin-7-yl)acetamide |  | C_13_H_26_BNO_6_Si  Amide | | 331.25 g/mol | | 80% |
|  | | | ***Enterobacter cloacae*** | | | | | |  |
| 1 | 8.86 | | Methyl hexadeca-7,9-dienoate |  | | [C_17_H_30_O_2_](https://pubchem.ncbi.nlm.nih.gov/#query=C17H30O2)  Ester | 266.4 g/mol | | 36.8% |
| 2 | 10.58 | | Pentadecanoic acid |  | | C_15_H_30_O_2_  Fatty acid | 242.3975 g/mol | | 33.3% |
| 3 | 10.73 | | n-Hexadecanoic acid |  | | C_16_H_32_O_2_  Fatty acid | 256.43 g/mol | | 53.8% |
| 4 | 11.38 | | 2-methylpentadecane-2-thiol |  | | [C_16_H_34_S](https://pubchem.ncbi.nlm.nih.gov/#query=C16H34S)  Thiol (Alcohol) | 258.5g/mol | | 72.7% |
| 5 | 11.99 | | 2-methylhexadecyl) holmium |  | | C_17_H_35_HO  Alcohol | 404.40 g/mol | | 62.5% |
| 6 | 14.39 | | 2-(2-chloro-4-methylphenoxy)-N, N-diethylethan-1-amine |  | | C_13_H_20_CINO  Amine | 241.76 g/mol | | 34.5% |
| 7 | 14.73 | | (1-ethyl-4-methyl-1,1a,2,3,4,4a,9,10 octahydrocyclopropa[3',4’] pyrido[2',3':3,4]cyclopenta[1,2-b]indol-10-yl)methyl acetate |  | | [C_21_H_26_N_2_O_2_](https://pubchem.ncbi.nlm.nih.gov/#query=C18H20N2O3)  Ester | 338.45 g/mol | | 47.8% |
| 8 | 15.50 | | (Z)-16-((Z)-2-hydroxy-1-(methoxyimino) ethyl)-10,13,17-trimethyl-4,15,16,17-tetrahydro-3H-10l5,13l5-cyclopenta[a]phenanthren-3-one O-methyl oxime |  | | C_24_H_30_NO_2_  Imine | 354.5 g/mol | | 63.1% |
|  | | | ***Bacillus subtilis*** | | | | | |  |
| 1 | 9.56 | | (E)-17-methoxy-10,13-dimethyl1,2,6,7,8,9,10,11,12,13,14,15,16,17-tetradecahydro-3H-cyclopenta[a]phenanthren-3-one O-methyl oxime |  | | [C_21_H_33_NO](https://pubchem.ncbi.nlm.nih.gov/#query=C8H9NO)_2_  Imine | 331.50 g/mol | | 36.8% |
| 2 | 12.02 | | N'-(3,6-dichloro-2,7-bis(2-(diethylamino) ethoxy)-9H-fluoren-9 ylidene)pivalohydrazide |  | | [C_29_H_40_Cl_2_N_4_O_3_](https://pubchem.ncbi.nlm.nih.gov/#query=C30H42Cl2N4O3)  Hydrazide | 563.56g/mol | | 71.4% |
| 3 | 14.39 | | 3-isobutylhexahydropyrrolo[1,2-a] pyrazine-1,4-dione |  | | C_11_H_18_N_2_O_2_  ketone | 210.273g/mol | | 65.6% |
| 4 | 15.52 | | 2,3-bis((trimethylsilyl)oxy) propyl (9Z,12Z)-9,12-octadecadienoate |  | | [C_27_H_54_O_4_Si_2_](https://pubchem.ncbi.nlm.nih.gov/#query=C27H54O4Si2)  Ester | 498.4g/mol | | 63.1% |
