# Supplementary figures and images for: Endophytic bacteria of Fagonia indica Burm. f revealed to harbour rich secondary antibacterial metabolites
Source: PLoS One. 2022 Dec 15;17(12):e0277825. doi: 10.1371/journal.pone.0277825 (PMC9754247; doi:10.1371/journal.pone.0277825)

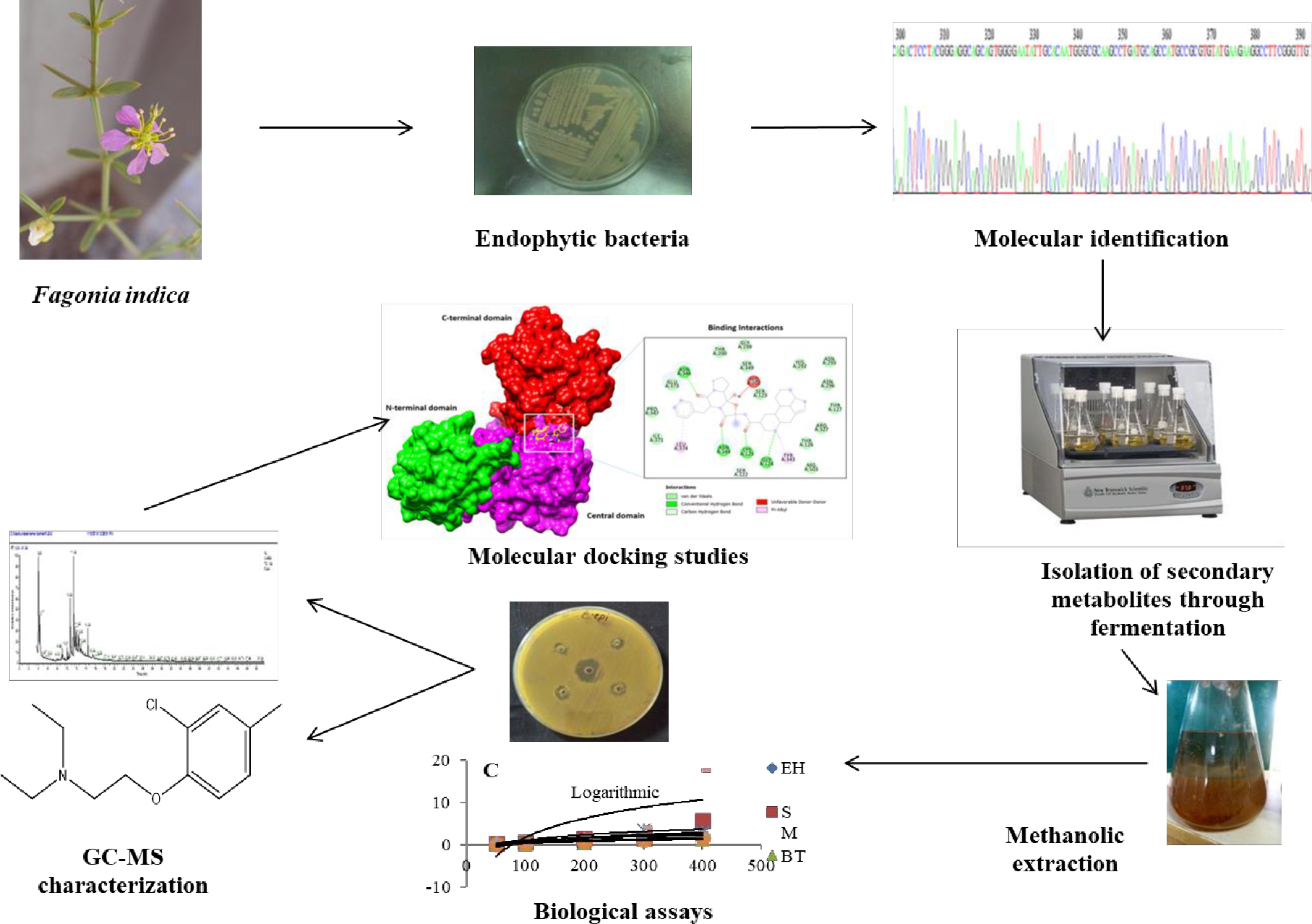

Supplement: S1 Graphical abstract — (TIF) [file pone.0277825.s005.tif]
